# Are sex differences in fundamental motor skills uniform throughout the entire preschool period?

**DOI:** 10.1371/journal.pone.0176556

**Published:** 2017-04-27

**Authors:** Jakub Kokštejn, Martin Musálek, James J. Tufano

**Affiliations:** Faculty of Physical Education and Sport, Charles University, Prague, Czech Republic; Boston Children's Hospital / Harvard Medical School, UNITED STATES

## Abstract

The aim of this study was to assess differences in fundamental motor skills (FMS) proficiency between boys and girls of each age group, independently, across the entire preschool period. Using the Movement Assessment Battery for Children–second edition, FMS proficiency was tested in 325 preschoolers (4.9 ± 1.1 y, range 3–6) using a cross-sectional design. Compared to boys of the same age, 3- and 4-year-old girls had greater total (p < .01), fine motor skill (p < .01), and balance scores (p < .05). There were no sex differences for total test or balance scores in 5- and 6-year-olds, but 6-year-old boys outperformed girls in aiming and catching (p < .001). These data not only agree with previous research in that sex differences in FMS proficiency exist in preschool children, but the data also show that differences may not be uniform throughout the whole preschool period when analyzing by age. To avoid under- or overestimating FMS proficiency and subsequently prescribing inaccurate motor intervention programs, FMS proficiency normative values should be age- and sex-specific throughout the entire preschool period.

## Introduction

During early childhood, children begin to learn and acquire many fundamental motor skills (FMS), which are commonly grouped into the functional skill categories of locomotor (e.g. running, hopping), balance (e.g. twisting, standing on one leg), and manipulative skills [1, 2]. Manipulative FMS are further divided into: 1) object control skills (e.g. catching, throwing, kicking) and 2) fine motor skills (e.g. sewing, cutting, and self-helping) that involve intricate use of the hand and wrist muscles [2].

Adequate acquisition and command of FMS at the end of early childhood (around the age of six) have been considered as crucial elements in the development of specialized and more complex motor skills in later years [1, 2, 3]. These are particularly important for children, not only for mastering habitual daily activities, but also for successful participation in both organized and non-organized sports and recreational activities [3].

Previous studies have shown that higher levels of FMS proficiency correlate with more physical activity [4–7] and greater physical fitness performance in preschool and school-aged children [8–10]. At the opposite end of the spectrum, poor FMS proficiency has been linked to delayed participation in physical activity, thus resulting in suboptimal levels of physical activity and fitness [10, 11]. Therefore, it is important to properly identify children who display FMS deficiencies in order to prescribe appropriate motor skill interventions to increase the likelihood of physical activity during adolescence.

Currently, FMS normative values include both sexes across four age categories for 3- and 4-year-olds (four six-month categories) as well as individual categories for 5- and 6-year-olds independently [12, 13]. Although a lack of sex-defining phenotypic gender characteristics often allow preschoolers of both sexes to be combined when describing physical development during early childhood, sex has been shown to play a role in acquiring and mastering FMS [14–17], sometimes as early as about three years of age [14].

Although preschool boys and girls generally do not differ in terms of total FMS test scores [18–20], object control skills have been shown to be similar between sexes [19, 20, 21], but have also been shown to be better in boys [16, 18, 22–25]. Locomotor skills have been shown to be similar between sexes [16, 19, 20, 22], better in girls [6, 18, 26], and better in boys [23]. Balance skills have shown to be similar between sexes [20, 27, 28] and better in girls [25, 29, 30]. Similarly, fine motor skills have been shown to be similar between sexes [20] and better in girls [17, 25, 29, 31]. Although the research process aims to reveal patterns that are repeatedly observed within a population in order to provide conclusive statements about a topic, the inconsistencies of the aforementioned body of literature do not allow for conclusive statements regarding FMS in preschool children.

Such discrepancies within the data can most likely be explained by a number of possibilities including: 1) studies not including children from the entire preschool period (3–6 years old); 2) studies often combining children of both sexes together; or 3) researchers creating two age group categories by combining 3- and 4-year-olds and 5- and 6-year-olds together. In fact, to the authors’ knowledge, boys and girls have not been compared against each other according to individual age groups since 1982 [32] where sex differences were present for locomotor and balance skills, but object control and fine motor skills were not investigated. Therefore, a detailed and up-to-date analysis of sex-specific FMS proficiency is needed in order to optimally develop FMS in modern day preschoolers.

To address this issue, the aim of this study was to compare FMS scores of preschool boys and girls for each age group across the entire preschool period. Based on the incongruous findings of the research discussed above, it was hypothesized that sex differences would occur in different types of FMS (manipulative, locomotor, and balance), but would not be uniform between boys and girls in each age group across the entire preschool period.

## Materials and methods

### Participants

Preschool children (n = 325: 162 boys and 163 girls; see Table 1) aged 3 to 6 years (4.9 ± 1.1 yrs) from seven randomly chosen preschools in two regions (Prague and central Bohemia) of the Czech Republic participated in the study. In cooperation with the management of the preschools, parents were informed of the purpose, procedures, and benefits of the study. The study was approved by the Ethics Committee of the Faculty of Physical Education and Sport at Charles University, Prague, and informed consent was required from the children’s parents or legal guardians. Children who had been diagnosed with mental or other clinically diagnosed impairments (such as ADHD, DCD, developmental dysphasia, etc.) and children from special needs classes were not included in the study.

**Table 1:** Numerical data of participants (N = 325).

Age		Boys	Girls
3 years	N	32	33
	Age (M±SD)	3.4±0.3	3.4±0.3
4 years	N	48	48
	Age (M±SD)	4.4±0.3	4.4±0.3
5 years	N	44	46
	Age (M±SD)	5.4±0.3	5.3±0.3
6 years	N	38	36
	Age (M±SD)	6.3±0.3	6.4±0.3

### Measures and procedures

The MABC-2 test AB1 (Movement Assessment Battery for Children-2 test Age Band 1) [12, 13] for preschool children assessed the level of FMS proficiency. The test contains eight test items which assess FMS proficiency in three basic motor domains: manual dexterity (MD), aiming and catching (AC), and balance (BAL) (Table 2).

**Table 2:** MABC-2 test for preschool children (version AB1).

MABC-2 test components	Task	Test Criterion
Manual dexterity (fine motor skills)	Posting Coins	Number of seconds
	Threading Beads	Number of seconds
	Drawing Trail	Number of errors
Aiming and Catching (gross motor skills)	Catching Beanbag	Number of correctly executed catches
	Throwing a Beanbag onto a Mat	Number of successful hits
Balance	One-Leg Balance	Number of seconds
	Walking Heels Raised	Number of correct consecutive steps
	Jumping on Mats	Number of correct consecutive jumps

A team of 4–6 trained research assistants (with Master’s degrees in Physical Education and Sport, Adapted Physical Education, Physiotherapy) tested children using the MABC-2 test AB 1. Before official testing and under the supervision of three MABC-2 certified testers, all examiners completed the user’s training program, which focused on understanding the theoretical issues and practical administration and scoring of the test. Research assistants performed the same tests for all children, meaning that there were no inter-rater testing procedures. Therefore, all children were scored by the same tester using the same guidelines.

Briefly, all testing occurred in a quiet kindergarten classroom during morning class time, and children were tested in small groups (2–3 children per group). The order of all eight tests was randomized and were performed on the same day. Children were familiarized with each test and performed two practice attempts for each. Then, children completed a single formal attempt, and the score of that attempt was used for data analysis.

The four motor tasks have different conditions between the younger (3- and 4-year old) and older (5- and 6-year old) children. For example, younger children must post six coins and thread six beads whereas older children must complete 12 of each. Younger children can use the entire body when catching a beanbag, but the older children must only use their hands. Lastly, young children can pause while jumping on mats, but older children are instructed to jump consecutively without pausing.

### Data analysis

Since raw scores from the MABC-2 test cannot and should not be compared between younger and older children due to the differences in testing protocols mentioned above, raw scores from each of the eight tests were converted to standard scores and percentiles in accordance with age-specific normative values for the Czech population [13]. The MABC manual allocates normative values for 5- and 6-year olds independently, while norms for 3- and 4-year olds are divided into two groups per age using 6-month categories, yielding four groups total (3 y 0 months to 3 y 6 months; 3 y 6 months to 4 y 0 months, etc.). For simplicity and clarity, these half-year categories were combined into a single score for each age, like the 5- and 6-year olds. The overall level of FMS was calculated by totaling the standard scores in the individual tests, and stated as the total test score (TTS) using percentile scores [13]. Scores for MD, AC and BAL were calculated in the same way. All data can be found in the S1 Dataset supplement file.

Percentile scores were used to interpret sex differences in TTS, MD, AC, and BAL for each age group (three to six years). Data normality was rejected, so the Mann-Whitney U test (*p* < .05) was used to determine statistical significance and the r coefficient was used to interpret effect size (ES), which can be interpreted as: *r* < 0.3 = small effect, *r* 0.3–0.5 = medium effect, and *r* > 0.5 = large effect [33]. Statistical analyses were conducted using the IBM SPSS Statistics 22 program.

## Results

**Fig 1:**
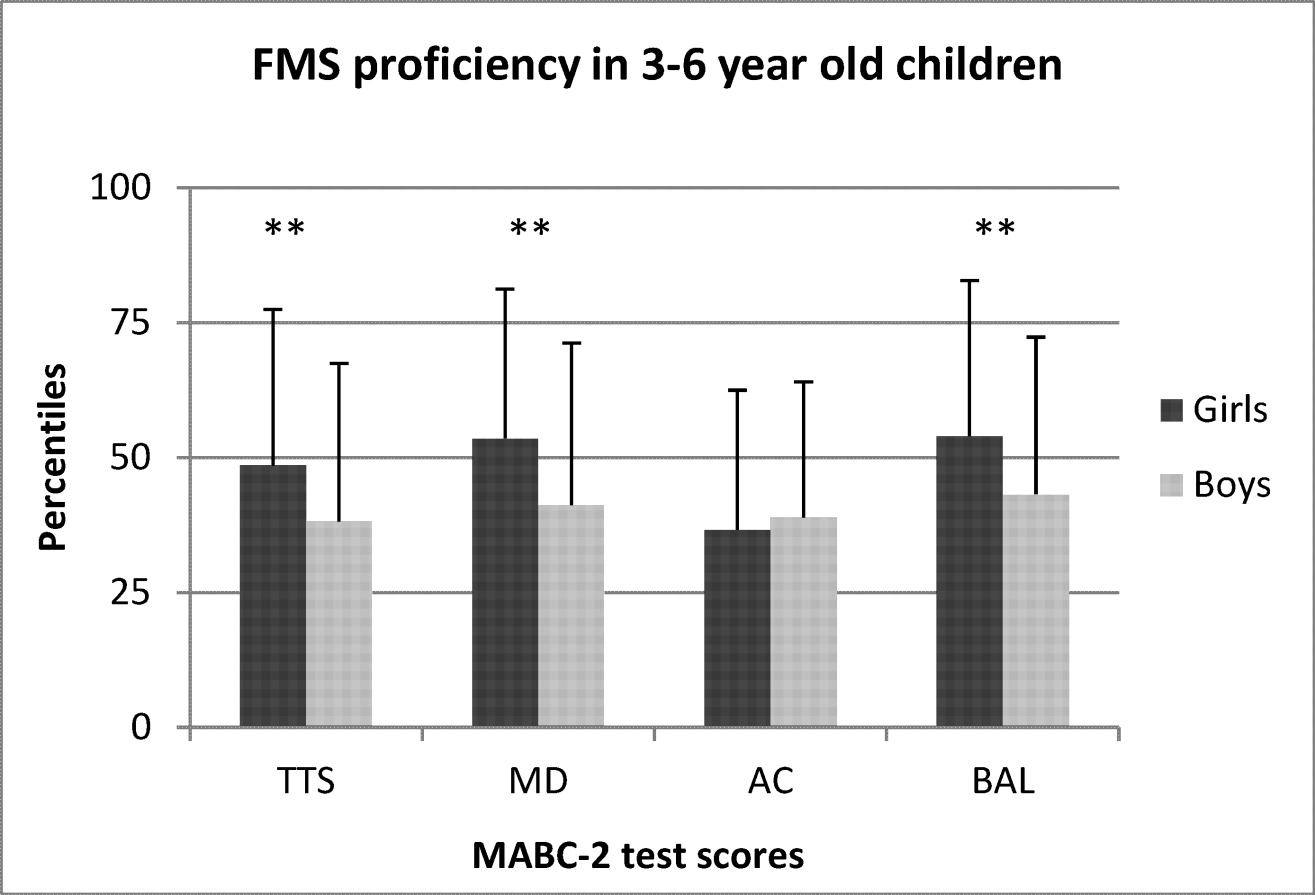
FMS proficiency in 3- to 6-year old children. Note: FMS = fundamental motor skills; MABC-2 = movement assessment battery for children-2; TTS = total test score; MD = manual dexterity; AC = aiming and catching; BAL = balance; ** p < .01.

**Fig 2:**
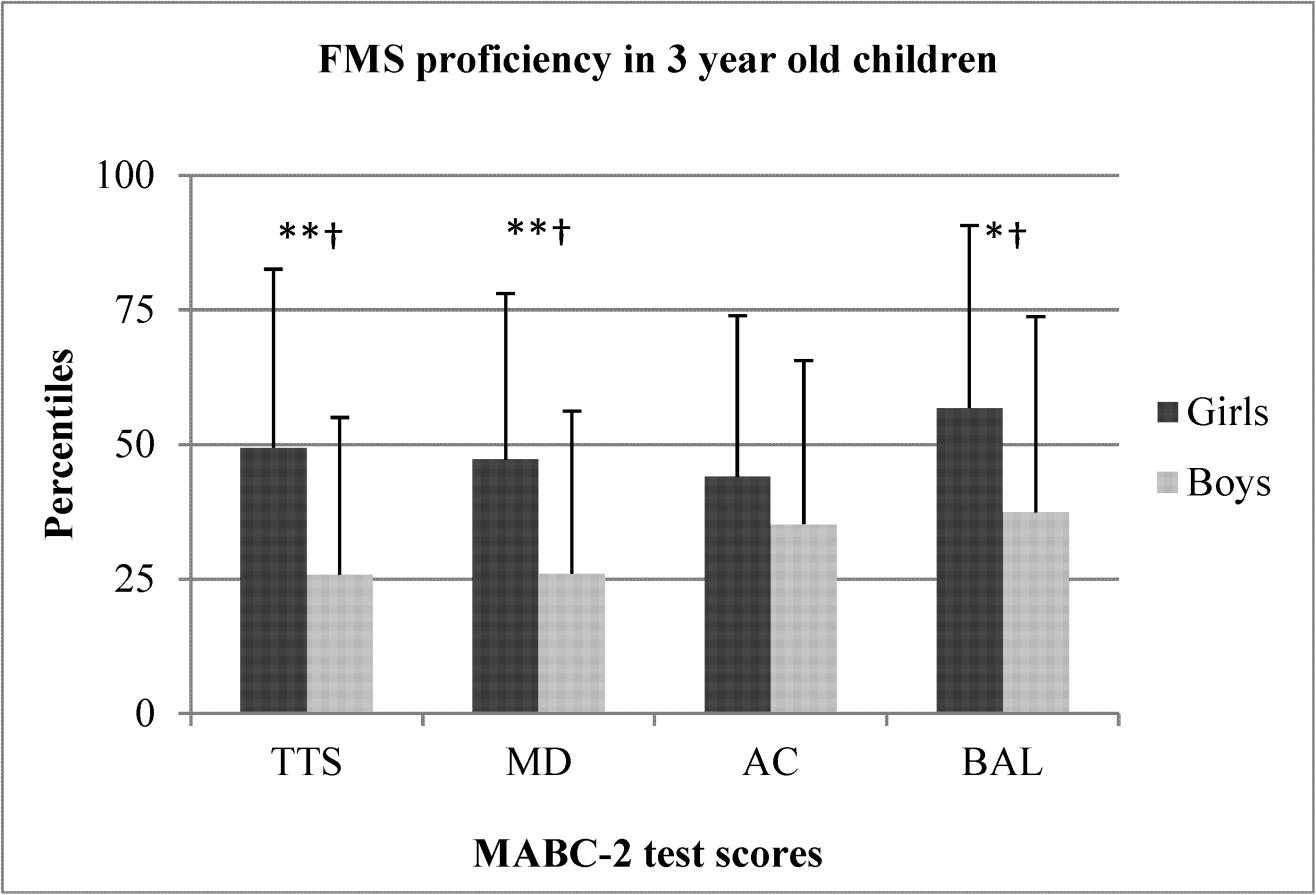
FMS proficiency in 3-year old children. Note: FMS = fundamental motor skills; MABC-2 = movement assessment battery for children-2; TTS = total test score; MD = manual dexterity; AC = aiming and catching; BAL = balance; ** p < .01; † medium effect size.

**Fig 3:**
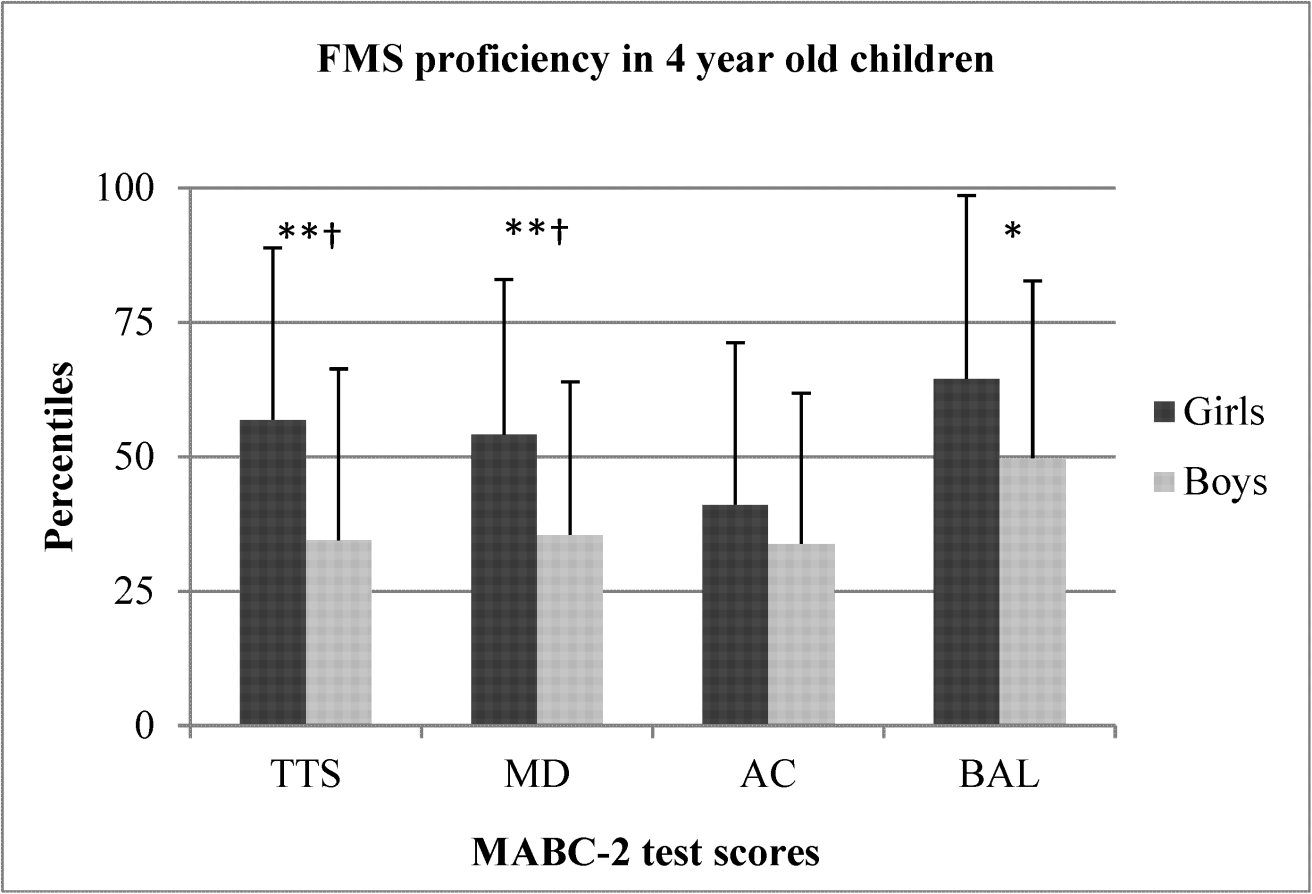
FMS proficiency in 4-year old children. Note: FMS = fundamental motor skills; MABC-2 = movement assessment battery for children-2; TTS = total test score; MD = manual dexterity; AC = aiming and catching; BAL = balance; ** p < .01; † medium effect size.

**Fig 4:**
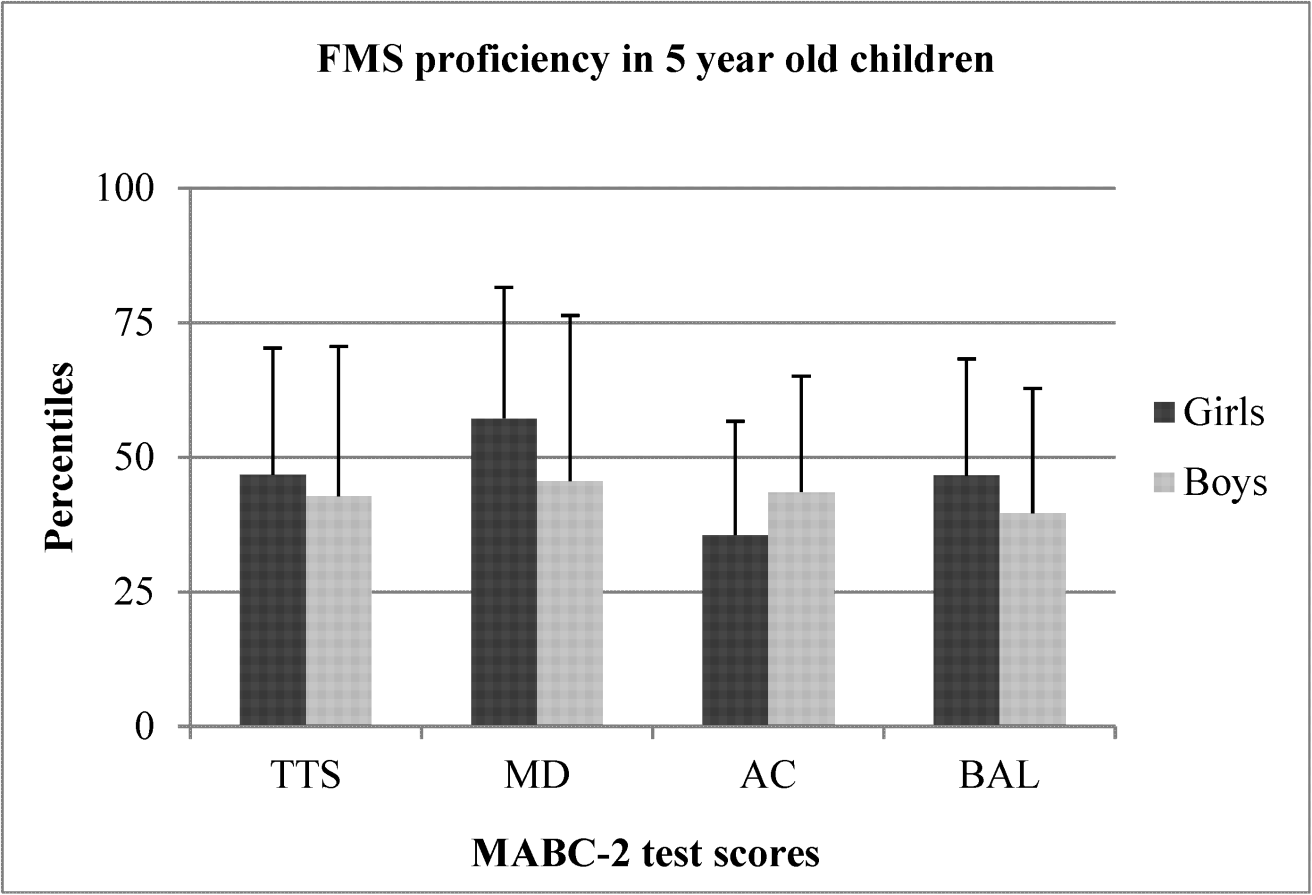
FMS proficiency in 5-year old children. Note: FMS = fundamental motor skills; MABC-2 = movement assessment battery for children-2; TTS = total test score; MD = manual dexterity; AC = aiming and catching; BAL = balance.

**Fig 5:**
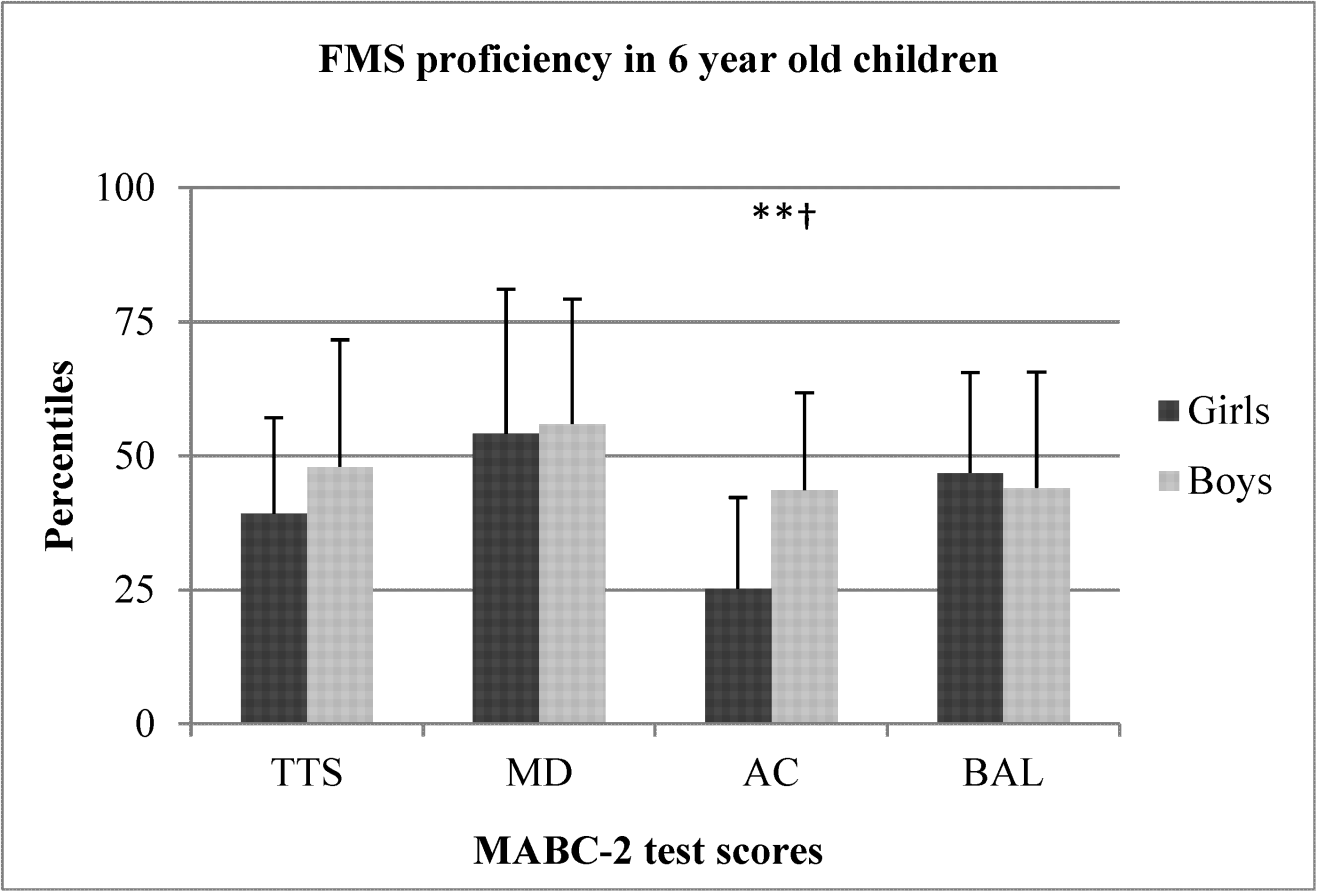
FMS proficiency in 6-year old children. Note: FMS = fundamental motor skills; MABC-2 = movement assessment battery for children-2; TTS = total test score; MD = manual dexterity; AC = aiming and catching; BAL = balance; *** p < .001; † medium effect size.

The sex differences between preschool boys and girls for TTS, MD, AC, and BAL are shown in Figs 1–5. When collapsed across age, girls had greater TTS (U = 10357.5 *p* < .01), MD (U = 9940 *p* < .01), and BAL (U = 23657 *p* < .01) scores compared to boys, but there were no differences in AC (Fig 1). At the age of 3, girls had greater TTS (U = 297.0 *p* < .01 ES = .38), MD (U = 306 *p* < .01; ES = .36), and BAL (U = 340.5 *p* < .05; ES = .31) scores than boys, but there were no differences in AC (Fig 2). At the age of 4, girls also scored higher than in TTS (U = 711.0 *p* < .01; ES = .33), MD (U = 738.0 *p* < .01; *r* = .31) and BAL (U = 861.5 *p* < .05), with no differences in AC (Fig 3). At the age of 5, there were no differences between sexes for any test (Fig 4). At the age of 6, there was also no difference in TTS, MD, and BAL between girls and boys, however, boys performed significantly better in the AC subtest of MABC-2 test (U = 306.5 *p* < .01; *r* = 0.48) (Fig 5). Small ES were not reported.

## Discussion

Generally, the results of previous studies do not allow practitioners to identify sex differences in FMS proficiency throughout the whole preschool period because studies either did not account for age [16, 19, 23, 24, 27], did not collect data on all age groups within the preschool period [4, 17, 18, 20, 21, 25, 27, 29–31, 34] or combined age groups together by comparing younger (3- and 4-year olds) and older (5- and 6-year olds) children [17, 22]. Therefore, an under- or overestimation of FMS competency in preschool children may be possible, resulting in inappropriate motor intervention programs. No study since 1982 [32] has investigated differences in FMS proficiency across the entire preschool period while also accounting for sex. To shed light on the FMS proficiency of modern-day children, the main aim of this study was to assess sex differences in FMS proficiency between boys and girls of all preschool ages. As hypothesized, there were significant sex differences in FMS proficiency in different motor domains, but the differences were not uniform throughout the entire preschool period when analyzed by age.

In the present study, younger girls (3–4 years old) outperformed boys in TTS, MD, and BAL, but no differences in AC were observed. In older children (5–6 years old), there were no differences in TTS, MD, and BAL, but 6-year old boys outperformed girls in AC. These data show that girls generally have better FMS scores than boys at a younger age and that these differences disappear toward the end of the preschool period. Perhaps more importantly, the average TTS and MD scores of 3-year old boys and AC score of 6-year old girls placed them all around the 25th percentile in their respective age groups [13]. Although speculative, it is unlikely that entire groups of children from preschools across an entire region performed worse than 75% of their peers. Rather, these data may suggest that there are maturational differences between sexes in preschoolers and that preschool boys and girls should not be compared to each other, but should be compared only to children of the same age and sex.

Previous studies using preschoolers of different cultural backgrounds have also noted that girls generally perform better than boys in TTS, MD, and BAL [25, 29, 34]. These results are in line with the results of Sigmundsson & Rostoff [29], who showed that 4-year old girls outperformed boys on TTS, MD, and BAL skills. However, their research sample only included 4-year olds, meaning that information about FMS proficiency in other preschool age categories was missing. Including children of all ages but not accounting for age, Kourtessis et al. [34] noted that girls outperformed boys in MD, and when using the same strategy, we also observed that girls performed better during MD tests (Fig 1). However, when assessing children according to sex and age, we found that only younger girls (3- and 4-year olds) scored better on MD tests than younger boys, but no sex differences were present in 5 or 6 year olds (Figs 4 and 5). Also accounting for sex and age, Livesey et al. [25] also showed that girls scored better on MD and BAL skills in 3- to 5-year old preschool children in Australia, leading the authors to state that it may be wise to create sex- and age-specific normative values for the MABC test. Additionally, our results agreed with previous studies in that 6-year old boys performed better than girls on AC [22, 35], but differences in AC skills did not exist between sexes in 3-, 4-, or 5-year olds. Based on our results, we support the idea of Livesey et al. [25] of separated norms for boys and girls on MABC-2 test, especially for younger preschool age children (3- and 4-year old).

To support this idea, it is plausible that differences in FMS proficiency between sexes exist during early childhood and can be credited to a complex interaction of environmental, socio-cultural, and biological factors [15, 36]. Specifically, it has been indicated that brain structure and development differs between sexes during infancy [37, 38], which may have residual effects during the toddler years, evidenced by enhanced development of the brain’s left hemisphere, which is mainly related to enhanced language acquisition, fine motor skills, and social cognition in young preschool girls compared to boys [39]. Thus, different sex-specific rhythms in brain maturation could suggest that 3- to 4-year old boys may need more time to develop fine motor skills and should not be compared to girls of the same age, supported by the seemingly sub-par performance of younger boys in nearly all FMS skills (Figs 2 and 3).

It is difficult to determine why 6-year old boys performed better than girls at throwing and catching in the present study. It has been hypothesized [15, 40–42] that environmental and socio-cultural factors may partly explain why preschool boys generally outperform girls at object control skills, as girls spend more time in language, literacy, art, and fine motor activities and boys in a number of different ball games and gross motor activities [40]. However, Thomas and French [15] had difficulty confirming this hypothesis after observing that boys were better at throwing in children as young as 3 years old, before environmental and socio-cultural factors could play a large role. Later, Nelson et al. [42] found sex differences in qualitative aspects in throwing skills of 5- to 9-year old children, and attributed the lower mastery of girls in that skill to a lack of practice and encouragement for girls in object control skills. As the present study utilized a cross-sectional design, meaning that age groups could not be compared to each other and patterns of FMS development could not be determined from the data, a longitudinal follow-up of children may allow for more accurate explanation as to why boys begin to outperform girls at object control skills as they get older. Lastly, the data presented should not be viewed as novel normative values, but instead indicate that preschool children of all ages and both sexes should not be compared to one another in the Czech Republic. Future researchers should determine if the same holds true in larger sample sizes across multiple countries.

Although MABC-2 norms exist, they are age-group specific and do not allow for the comparison of FMS between age groups. The data from this study provide evidence that FMS proficiency differs between sexes in preschool children, but that these differences are not uniform through a whole age categories when analyzing by year of age. Therefore, in order to successfully identify children whom lack FMS proficiency, sex- and age-specific normative values for each FMS test should be created to allow for more appropriate, multifaceted, and individualized motor intervention programs for developing FMS proficiency in preschoolers.

## Supporting information

S1 Dataset (XLSX)
